# Supplementary material for: Serious Underlying Medical Conditions and COVID-19 Vaccine Hesitancy: A Large Cross-Sectional Analysis from Australia
Source: Vaccines (Basel). 2022 May 26;10(6):851. doi: 10.3390/vaccines10060851 (PMC9230066; doi:10.3390/vaccines10060851)

Supplementary materials

These supplementary materials have been provided by the authors to give readers additional information about their work

Supplement to: Serious Underlying Medical Conditions and COVID-19 Vaccine Hesitancy: A Large Cross-sectional Analysis From Australia.

Table of contents

List of CANVACCS, DIABVACCS, and MSVACCS investigators....2

Table S1. Chronic disease COVID-19 Vaccine Survey Items. 3

Table S2. Participant characteristics, by vaccination status and disease. 8

Figure S1. Response frequencies for each Disease Influenced Vaccine Acceptance Scale-Six item, by chronic disease type and vaccination status (a-f). 'Don't know' responses have been excluded. 9

List of CANVACCS, DIABVACCS, and MSVACCS investigators

Study Site, Australian State	Investigator(s)
**Monash Health, Victoria**	
CANVACCS	Veronica Lopez Aedo
	Elizabeth Ahern
	Muhammad Alamgeer
	Nathan Bain
	Amy Body
	Peter Briggs
	Daphne Day
	Sophia Frentzas
	Lisa Grech
	Marion Harris
	Gwo-Yaw Ho
	Caroline Lum
	Vi Luong
	Amelia McCartney
	Cameron McLaren
	Mike Nguyen
	Stephen Opat
	David Pook
	Eva Segelov
	Andrew Strickland
	Avraham Travers
	Kate Webber
	Michelle White
	Walid Zwieky
DIABVACCS	Barbora de Courten
	Jennifer Wong
MSVACCS	Michelle Allan
	Ernest Butler
**Bendigo Health, Victoria**	
CANVACCS	Sam Harris
DIABVACCS	Frank Gao
	Amy Harding
	Mark Savage
**Latrobe Regional Hospital, Victoria**	
CANVACCS	Hieu Chau
**Sunshine Coast Hospital and Health Service, Queensland**	
CANVACCS	Bryan Chan
DIABVACCS	Brett Sillars
MSVACCS	Joshua Barton
	Antony Winkel
**Icon Cancer Centre Hobart, Tasmania**	
CANVACCS	Louise Nott
**Central Coast Haematology, New South Wales**	
CANVACCS	Richard Blennerhassett
	Cecily Forsyth
	Jacqueline Jagger
**St Vincent's Hospital Sydney, New South Wales**	
CANVACCS	Nada Hamad
**Campbelltown Hospital, New South Wales**	
CANVACCS	Annette Tognela
**Dr David Hoffman, New South Wales**	
DIABVACCS	David Hoffman
**Border Medical Oncology, New South Wales**	
CANVACCS	Craig Underhill

Abbreviations: CANVACCS, CANcer patients' perspectives on coronavirus VACCination Survey; DIABVACCS, DIABetes patients' perspectives on coronavirus VACCination Survey; MSVACCS, Multiple Sclerosis patients' perspectives on coronavirus VACCination Survey.

Table S1. Chronic disease COVID-19 Vaccine Survey Items.

**Screening items**		
1.	Are you 18 years or older?	o Yes<br>o No (Terminate if No)
2.	Have you got [chronic disease]?	o Yes<br>o No (Terminate if No)
3.	Are you a [participating site] patient?	o Yes<br>o No (Terminate if No)
**Vaccination status**		
4.	Have you already received a COVID-19 vaccine?	o Yes, 1 dose only<br>o Yes, 2 doses<br>o No
**Oxford COVID-19 Vaccine Hesitancy Scale**<br>Instructions: We would like to know your feelings and thoughts about the COVID-19 vaccine. Note: If you have already been vaccinated against COVID-19, please complete these questions in relation to a future COVID-19 vaccine dose/booster.		
5.	Would you take a COVID-19 vaccine if offered?	o Definitely/have taken<br>o Probably<br>o I may or may not<br>o Probably not<br>o Definitely not<br>o Don't know
6.	When a COVID-19 vaccine is available:	o I will want to get it as soon as possible<br>o I will take it when offered<br>o I'm not sure what I will do<br>o I will put off (delay) getting it<br>o I will refuse to get it<br>o Don't know
7.	I would describe my attitude towards receiving a COVID-19 vaccine as:	o Very keen<br>o Pretty positive<br>o Neutral<br>o Quite uneasy<br>o Against it<br>o Don't know
8.	If a COVID-19 vaccine was available in my local area, I would:	o Get it as soon as possible<br>o Get it when I have time<br>o Delay getting it<br>o Avoid getting it for as long as possible<br>o Never get it<br>o Don't know
9.	If my family or friends were thinking of getting a COVID-19 vaccination, I would:	o Strongly encourage them<br>o Encourage them<br>o Not say anything to them about it<br>o Ask them to delay getting the vaccination<br>o Suggest that they do not get the vaccination<br>o Don't know
10.	I would describe myself as:	o Eager to get a COVID-19 vaccine<br>o Willing to get the COVID-19 vaccine<br>o Not bothered about getting the COVID-19 vaccine<br>o Unwilling to get the COVID-19 vaccine<br>o Anti-vaccination for COVID-19<br>o Don't know
11.	Taking a COVID-19 vaccination is:	o Really important<br>o Important<br>o Neither important nor unimportant<br>o Unimportant<br>o Really unimportant<br>o Don't know
**Oxford COVID-19 Vaccine Confidence and Complacency Scale**		
12.	Do you think you will be infected with COVID-19 over the next 12 months?	o Definitely<br>o Probably<br>o Possibly<br>o Probably not

	o Definitely not<br>o Don’t know
13. I think the COVID-19 vaccine is likely to:	o Work for almost everyone<br>o Work for most people<br>o I am unsure how many people it will work for<br>o Not work for most people<br>o Not work for anyone<br>o Don’t know
14. I think the COVID-19 vaccine is likely to:	o Definitely work for me<br>o Probably work for me<br>o May or may not work for me<br>o Probably not work for me<br>o Definitely not work for me<br>o Don’t know
15. I think if I get the COVID-19 vaccine it will be:	o Really helpful for the community around me<br>o Helpful for the community around me<br>o Neither helpful nor unhelpful for the community around me<br>o Unhelpful for the community around me<br>o Really unhelpful for the community around me<br>o Don’t know
16. I think if individuals like me get the COVID-19 vaccine it will:	o Save a large number of lives<br>o Save some lives<br>o Have no impact<br>o Lead to more deaths<br>o Lead to a large number of deaths<br>o Don’t know
17. I think the speed of developing and testing the vaccine means it will be:	o Really good<br>o Good<br>o Will not affect how good or bad it is<br>o Bad<br>o Really bad<br>o Don’t know
18. I think the speed of developing and testing the vaccine means it will be:	o Really safe<br>o Safe<br>o It will not affect how safe it is<br>o Unsafe<br>o Really unsafe<br>o Don’t know
19. I think if many people do not get the vaccine this:	o Will be dangerous<br>o May be dangerous<br>o Will have no consequences at all<br>o May be good<br>o Will be good<br>o Don’t know
20. I expect that receiving the vaccine will be:	o Hardly noticeable<br>o A little unpleasant<br>o Moderately unpleasant<br>o Painful<br>o Extremely painful<br>o Don’t know
21. I think the side-effects for people of getting the COVID-19 vaccine will be:	o None<br>o Mild<br>o Moderate<br>o Significant<br>o Life-threatening<br>o Don’t know
22. I think the COVID-19 vaccine will:	o Greatly strengthen my immune system<br>o Strengthen my immune system<br>o It will neither strengthen nor weaken my immune system<br>o Weaken my immune system<br>o Greatly weaken my immune system<br>o Don’t know
23. I think taking the COVID-19 vaccine:	o Will give me complete freedom to get on with life just as before<br>o Will give me greater freedom

	o Will have no effect on my freedom<br>o Will restrict my freedom<br>o Will completely restrict my freedom to get on with life<br>o Don't know
24. I think getting the vaccine is a sign of:	o Great personal strength<br>o Personal strength<br>o Not a sign of personal strength or weakness<br>o Personal weakness<br>o Great personal weakness<br>o Don't know
25. Taking a new COVID-19 vaccine will make me feel like a guinea pig:	o Do not agree<br>o Agree a little<br>o Agree moderately<br>o Agree a lot<br>o Completely agree<br>o Don't know
**Disease Influenced Visual Acceptance Scale-Six**<br>Instructions: We would like to know about how your [chronic disease] may be related to your feelings and thoughts about the COVID-19 vaccine. For each of the following statements, please tap/click the one choice that best represents how strongly you agree or disagree with it. There are 6 choices to choose from for each statement.	
26. My history of [chronic disease] makes me more worried about being infected with COVID-19:	o Strongly agree<br>o Somewhat agree<br>o Neither disagree nor agree<br>o Somewhat disagree<br>o Strongly disagree<br>o Don't know
27. My history of [chronic disease] means having the vaccine is more important to me:	o Strongly agree<br>o Somewhat agree<br>o Neither disagree nor agree<br>o Somewhat disagree<br>o Strongly disagree<br>o Don't know
28. My doctor's recommendation regarding the vaccine is important to me:	o Strongly agree<br>o Somewhat agree<br>o Neither disagree nor agree<br>o Somewhat disagree<br>o Strongly disagree<br>o Don't know
29. My history of [chronic disease] makes me worried about how well the vaccine will work for me:	o Strongly disagree<br>o Somewhat disagree<br>o Neither disagree nor agree<br>o Somewhat agree<br>o Strongly agree<br>o Don't know
30. My history of [chronic disease] makes me worried about how the vaccine will affect me:	o Strongly disagree<br>o Somewhat disagree<br>o Neither disagree nor agree<br>o Somewhat agree<br>o Strongly agree<br>o Don't know
31. I am worried about how the vaccine will affect my [chronic disease] treatment:	o Strongly disagree<br>o Somewhat disagree<br>o Neither disagree nor agree<br>o Somewhat agree<br>o Strongly agree<br>o Don't know
**Demographics**	
32. What is your gender?	o Male<br>o Female<br>o Non-binary / third gender<br>o Prefer not to say
33. What is your age?	________________

34.	What is your highest educational level (completed)?	o No formal education<br>o Primary education<br>o Secondary education<br>o Vocational/trade qualification<br>o University education or higher degree<br>o Other (please specify): _____________________
35.	What is your annual household income (including everyone who lives in your home)?	o Less than $50,000<br>o $50,001 to $100,000<br>o $100,001 to $150,000<br>o More than $150,000<br>o Prefer not to say
36.	Do you identify as Aboriginal and/or Torres Strait Islander?	o Yes<br>o No<br>o Prefer not to say
37.	Is English your first language?	o Yes<br>o No
38.	Please include any comments about your feelings and thoughts about your [chronic disease] and COVID-19 vaccination that you would like to share. If you have no comments to include, please type 'Nil'.	[Text entry]: _______________________________________
**Disease-specific clinical questions (Cancer only)**		
39.	What type of cancer do you have? (i.e. where your cancer started and not where it has spread to).	o Breast<br>o Lung (including mesothelioma)<br>o Genitourinary (i.e. prostate, kidney, testicular or bladder)<br>o Skin (including melanoma)<br>o Gastrointestinal (i.e. stomach, oesophagus, bile duct, gallbladder, pancreas, colon, rectum, or anus)<br>o Gynaecological (i.e. ovarian, cervical, uterine or vulvar/vaginal)<br>o Head and neck (i.e. mouth, throat, sinus or nose)<br>o Brain<br>o Blood (i.e. leukemia, myeloma and lymphoma)<br>o Other (please specify):___________________________
40.	When was your cancer diagnosed?	o Less than 6 months ago<br>o 6 to 24 months ago<br>o 2 to 5 years ago<br>o More than 5 years ago
41.	As you understand it, is your cancer in just the one area where it started (localised) or has it spread to other places in the body (metastatic)?	o Localised<br>o Metastatic<br>o Don't know<br>o Other (please type any comments): _____________________
42.	Are you currently on cancer treatment?	o Yes<br>o No
43.	How long ago was your last treatment? (e.g. chemotherapy, immunotherapy, hormonal treatment, targeted therapy, radiotherapy and/or clinical trial).	o Currently on treatment<br>o Less than 1 year ago<br>o 1 to 5 years ago<br>o More than 5 years ago
**Disease-specific clinical questions (Diabetes only)**		
39.	What type of diabetes do you have?	o Type 1<br>o Type 2<br>o Other (please specify): _________________________________<br>o Don't know
40.	How long have you had diabetes?	o Less than 1 year<br>o 1 to 5 years<br>o 5.1 to 10 years<br>o More than 10 years
41.	My most recent HbA1c (within the past year) was:	o Less than 7%<br>o 7% to 8.5%<br>o 8.6% to 10%<br>o More than 10%<br>o Don't know

42.	My current treatment for my diabetes is/are (select all that apply):	o Insulin<br>o Tablets<br>o Diet only<br>o Injectables (not insulin)<br>o Other: ________________________________
43.	In the past month, would you say that your management of diabetes was:	o Excellent<br>o Very good<br>o Good<br>o Fair<br>o Poor
44.	In the last four weeks, how much did your diabetes affect your daily activities?	o All the time<br>o Most of the time<br>o Some of the time<br>o Not very often<br>o Not at all
**Disease-specific clinical questions (Multiple Sclerosis only)**		
39.	What type of MS do you have?	o Relapsing-remitting MS (RRMS)<br>o Primary progressive MS (PPMS)<br>o Secondary progressive MS (SPMS)<br>o Other (please specify): ________________________________<br>o Don't know
40.	How long have you had MS?	o Less than 1 year<br>o 1 to 5 years<br>o 5.1 to 10 years<br>o More than 10 years
41.	My current treatment for my MS is/are (select all that apply):	o Tablets<br>o Injectables<br>o Intravenous<br>o No specific treatment<br>o Other: ______________________________________
42.	Over the past 6 months, is your MS well controlled?	o Yes<br>o No<br>o Don’t know
43.	People often have difficulty taking their medications for one reason or another. How many times have you missed taking your disease modifying therapies (DMT) in the past month?	o All of the time<br>o Most of the time<br>o Some of the time<br>o Occasionally<br>o Never
44.	In the last four weeks, how much did your MS affect your daily activities?	o All the time<br>o Most of the time<br>o Some of the time<br>o Not very often<br>o Not at all

Table S2. Participant characteristics, by vaccination status and disease.

	All diseases n, (%)				Cancer n, (%)				Diabetes n, (%)				MS n, (%)			
**Characteristics**	**Vaccinated (n = 3813)**	**Unvaccinated (n = 868)**	**p[a]**	**φ[b]**	**Vaccinated (n = 2884)**	**Unvaccinated (n = 674)**	**p[a]**	**φ[b]**	**Vaccinated (n = 696)**	**Unvaccinated (n=146)**	**p[a]**	**φ[b]**	**Vaccinated (n = 233)**	**Unvaccinated (n = 48)**	**p[a]**	**φ[b]**
**Gender**			0.002	0.05			0.004	0.05			0.37	0.03			0.35	0.07
Male	1758 (83.5)	348 (16.5)			1318 (83.2)	266 (16.8)			383 (83.8)	74 (16.2)			57 (87.7)	8 (12.3)		
Female	2034 (79.8)	514 (20.2)			1553 (79.4)	404 (20.6)			307 (81.2)	71 (18.8)			174 (81.7)	39 (18.3)		
Non-Binary/Other	21 (77.8)	6 (22.2)			13 (76.5)	4 (23.5)			6 (85.7)	1 (14.3)			2 (66.7)	1 (33.3)		
**Age**			<0.001[c]	0.03[c]			<0.001[c]	0.04[c]			<0.001[c]	0.03[c]			<0.001[c]	0.06[c]
Mean (SD)	61.8 (13.0)	55.6 (13.5)			64.1 (11.7)	57.9 (12.3)			56.3 (14.3)	49.8 (14.6)			49.1 (12.6)	40.6 (11.7)		
18 – 29	79 (66.4)	40 (33.6)			13 (52.0)	12 (48.0)			47 (72.3)	18 (27.7)			19 (65.5)	10 (34.5)		
30 – 49	576 (72.6)	217 (27.4)			341 (70.3)	144 (29.7)			141 (75.8)	45 (24.2)			94 (77.0)	28 (23.0)		
50 – 69	1953 (80.1)	486 (19.9)			1458 (78.2)	406 (21.8)			384 (84.4)	71 (15.6)			111 (92.5)	9 (7.5)		
≥70	1202 (90.6)	125 (9.4)			1069 (90.5)	112 (9.5)			124 (91.2)	12 (8.8)			9 (90.0)	1 (10.0)		
**Highest level of education**			<0.001	0.07[d]			<0.001	0.07[d]			0.37	0.06[d]			0.23	0.12[d]
No formal/ Primary	96 (73.3)	35 (26.7)			66 (73.3)	24 (26.7)			27 (73.0)	10 (27.0)			3 (75.0)	1 (25.0)		
Secondary	1272 (81.0)	298 (19.0)			931 (79.9)	234 (20.1)			268 (84.0)	51 (16.0)			73 (84.9)	13 (15.1)		
Vocational/Trade	951 (79.1)	251 (20.9)			705 (78.7)	191 (21.3)			189 (81.8)	42 (18.2)			57 (76.0)	18 (24.0)		
University	1487 (84.3)	277 (15.7)			1175 (84.2)	221 (15.8)			212 (83.8)	41 (16.2)			100 (87.0)	15 (13.0)		
Other	7 (50.0)	7 (50.0)			7 (63.6)	4 (36.4)			0 (0.0)	2 (100.0)			0 (0.0)	1 (100.0)		
**Annual household income (AUD)**			<0.001	0.08[d]			<0.001	0.09[d]			0.04	0.11[d]			0.58	0.10[d]
<50,000	1260 (81.4)	288 (18.6)			915 (79.4)	238 (20.6)			283 (87.6)	40 (12.4)			62 (86.1)	10 (13.9)		
50,000 - 100.000	946 (82.5)	201 (17.5)			712 (83.4)	142 (16.6)			175 (80.5)	42 (19.4)			59 (77.6)	17 (22.4)		
100.000 - 150,000	466 (78.3)	129 (21.7)			363 (78.6)	99 (21.4)			70 (76.1)	22 (23.9)			33 (80.5)	8 (19.5)		
>150,000	489 (87.9)	67 (12.1)			412 (88.8)	52 (11.2)			42 (80.8)	10 (19.2)			35 (87.5)	5 (12.5)		
Prefer not to say	652 (78.1)	183 (21.9)			482 (77.1)	143 (22.9)			126 (79.7)	32 (20.3)			44 (84.6)	8 (15.4)		
**English as primary language**			<0.001	0.07			<0.001	0.11			0.24	-0.04			0.43	0.06
Yes	3437 (82.4)	735 (17.6)			2668 (82.4)	570 (17.6)			554 (81.8)	123 (18.2)			215 (83.7)	42 (16.3)		
No	376 (74.0)	132 (26.0)			216 (67.7)	103 (32.3)			142 (86.1)	23 (13.9)			18 (75.0)	6 (25.0)		
**Location**			<0.001	0.06			<0.001	0.08			0.60	-0.02			0.14	0.10
Metropolitan location	2686 (83.1)	548 (16.9)			1987 (83.1)	403 (16.9)			497 (82.1)	108 (17.9)			202 (84.5)	37 (15.5)		
Regional/Rural location	1127 (77.9)	320 (22.1)			897 (76.8)	271 (23.2)			199 (84.0)	38 (16.0)			31 (73.8)	11 (26.2)		

Abbreviations: AUD, Australian Dollars.

[a] Chi-square p-value for differences between vaccination status. Chi-square analyses for gender did not include non-binary/other categories and for highest educational level, it did not include the other category.

[b] Phi coefficient.

[c] Independent samples t-test p-value and eta squared reported, as they were used to measure differences between vaccination status and effect size for age as a continuous variable, respectively.

[d] Cramer's V reported.

Significant differences between vaccinated and not vaccinated for patients who identify as Aboriginal and/or Torres Strait Islander were detected only for: 1) all diseases (p<0.001) and 2) cancer (p=0.003).

Figure S1. Response frequencies for each Disease Influenced Vaccine Acceptance Scale-Six item, by chronic disease type and vaccination status (a-f). 'Don't know' responses have been excluded.

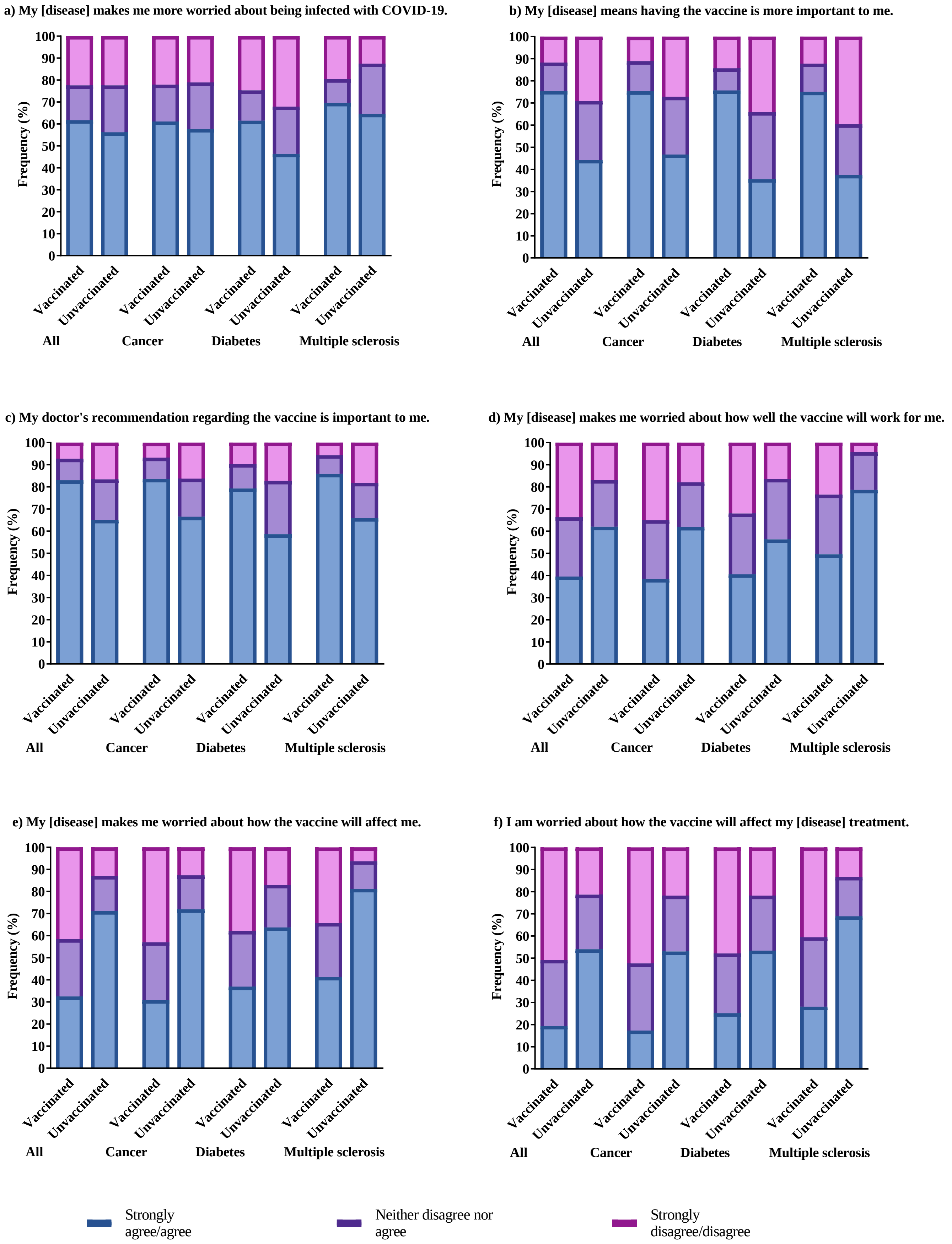

Supplement: Supplementary file 1 [file vaccines-10-00851-s001.zip › vaccines-1741637-supplementaryV2.pdf]
